# Supplementary material for: ApoE4 disrupts interaction of sortilin with fatty acid-binding protein 7 essential to promote lipid signaling
Source: J Cell Sci. 2021 Oct 21;134(20):jcs258894. doi: 10.1242/jcs.258894 (PMC8572006; doi:10.1242/jcs.258894)
Supplement: Supplementary information [file joces-134-258894-s1.pdf]

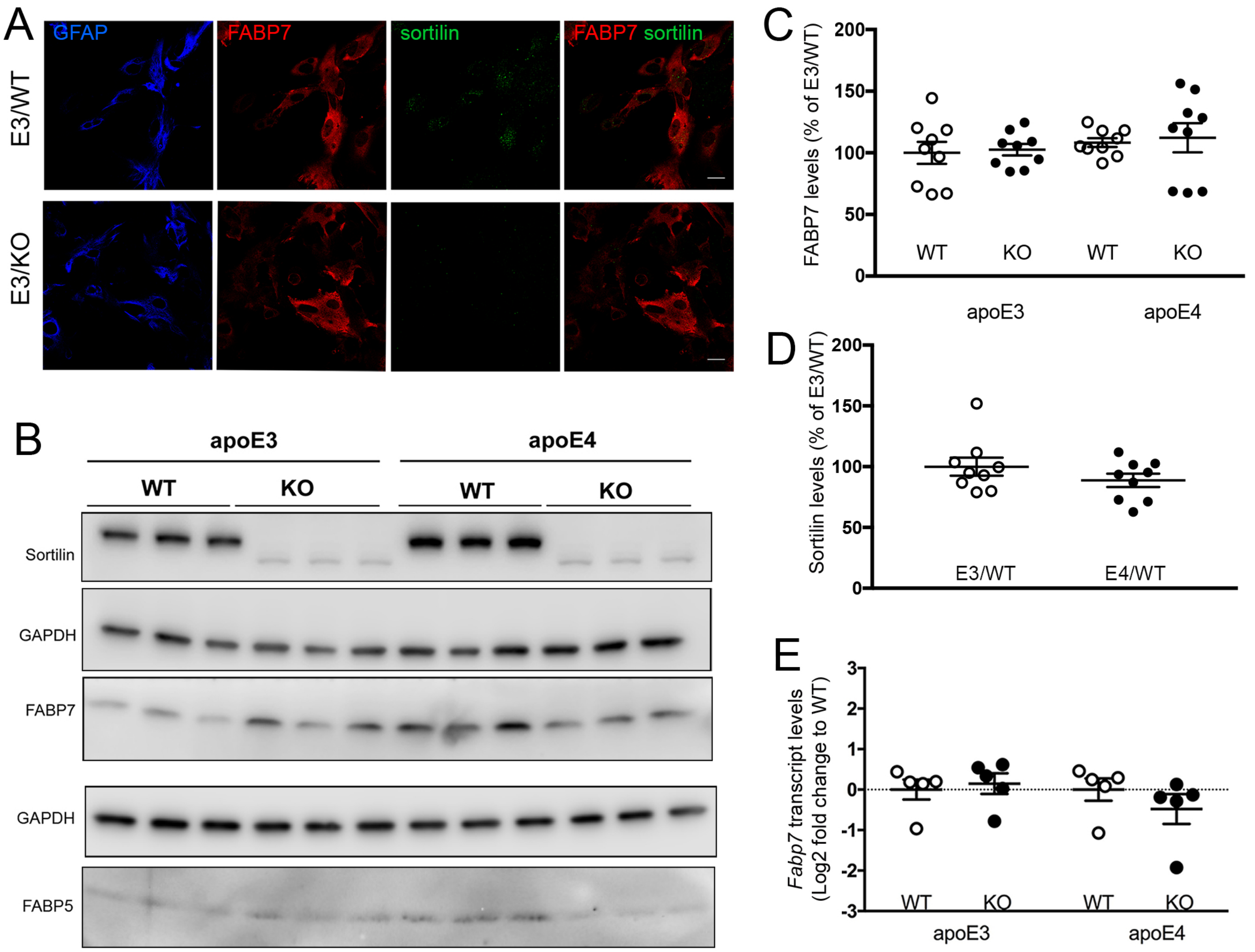

Asaro et al., Figure S1

Journal of Cell Science • Supplementary information

Fig. S1. ***APOE*** **genotype does not impact levels of sortilin or FABP7 in primary astrocytes**

(**A**) Immunodetection of FABP7 (red) and sortilin (green) in primary astrocytes from apoE3 mice either (WT) or genetically deficient for *Sort1* (KO). FAPB7-stained cells are identified as astrocytes by expression of GFAP (blue). Merged images show co-expression of FABP7 and sortilin. Images represent single z-planes. Scale bar: 20 µm. (**B**) Exemplary western blot analyses of sortilin, FABP5, and FABP7 in primary astrocytes from apoE3 or apoE4 targeted replacement mice, either wild-type (WT) or homozygous for the *Sort1* null allele (KO). Detection of GAPDH served as loading control. (**C - D**) Quantitative analysis of FABP7 (C) and sortilin (D) levels in primary astrocyte cultures using densitometric scanning of replicate blots (as exemplified in panel B). No genotype-dependent alterations in FABP7 or sortilin levels were seen comparing cells of the indicated *APOE* and *Sort1* genotypes. Data are given as mean ± SEM with E3/WT levels set to 100% (n=9 independent cultures per genotype). (**E**) Levels of *Fabp7* transcript were identical in primary astrocytes from apoE3- and apoE4-expressing mice, either WT or KO for *Sort1*, as determined by quantitative RT-PCR (n=5 independent cultures per genotype). Values are given as $\log_2$ fold change relative to the respective WT (set to value 0).

Journal of Cell Science • Supplementary information

Table S1. List of selected proteins with altered cell surface localization in primary cultures of sortilin-deficient neurons or astrocytes

ACC	Protein	Gene	ratio (KO/WT)	*p*-value
		**Neurons**		
P15209	BDNF/NT-3 growth factors receptor	*Trkb*	0.724	0.020768
Q01279	Epidermal growth factor receptor	*Egfr*	0.793	0.000107
Q04519	Sphingomyelin phosphodiesterase	*Asmase*	1.53	0.005667
Q8C0E2	Vacuolar protein sorting-associated protein 26B	*Vps26b*	1.56	0.0035004
P51880	Fatty acid-binding protein, brain (FABP7)	*Fabp7*	1.52	0.033944
P11404	Fatty acid-binding protein, heart (FABP3)	*Fabp3*	1.4	0.22835
Q05816	Fatty acid-binding protein, epidermal (FABP5)	*Fabp5*	1.25	0.11495
		**Astrocytes**		
P15209	BDNF/NT-3 growth factors receptor	*Trkb*	0.774	0.03436
P28798	Progranulin	*Grn*	0.646	0.000474
Q06335	Amyloid-like protein 2	*Aplp2*	0.707	0.000877
P41233	ATP-binding cassette transporter ABCA1	*Abca1*	0.756	0.003095
P51880	Fatty acid-binding protein, brain (FABP7)	*Fabp7*	0.94	0.61984
P11404	Fatty acid-binding protein, heart (FABP3)	*Fabp3*	not detected	
Q05816	Fatty acid-binding protein, epidermal (FABP5)	*Fabp5*	not detected	

ACC, Uniprot accession number; ratio (KO/WT), ratio between protein amounts in cell surface fractions from sortilin KO and WT primary cell cultures as determined by quantitative label-free proteomics; *p*-value is given based on a resampling ANOVA-based significance test.

Table S2. Patient samples examined by western blot analysis. Specimens were obtained from the Netherlands Brain Bank (NL; Netherlands Institute for Neuroscience, Amsterdam) and the MRC London Brain Bank for Neurodegenerative Diseases (UK; Institute of Psychiatry, King's College London).

Case No.	Age	Sex	Clinical diagnosis	Braak stage	ApoE Genotype	Brain Bank
94-110	82	F	AD dementia	VI	4/4	NL
94-037	63	M	AD dementia	VI	4/4	NL
94-082	64	M	AD dementia	VI	4/4	NL
98-060	66	M	AD dementia	VI	4/4	NL
94-091	83	F	AD dementia	VI	3/3	NL
94-025	85	F	AD dementia	VI	3/3	NL
94-016	86	F	AD dementia	VI	3/3	NL
93-140	87	F	AD dementia	IV	3/3	NL
97-009	89	F	AD dementia	VI	3/3	NL
92-022	91	F	AD dementia	IV	3/3	NL
93-011	92	F	AD dementia	VI	3/3	NL
99-114	92	F	AD dementia	IV	3/3	NL
99-123	93	F	AD dementia	IV	3/3	NL
96-020	58	M	AD dementia	VI	3/3	NL
94-028	70	M	AD dementia	VI	3/3	NL
95-077	72	M	AD dementia	VI	3/3	NL
90-066	89	M	AD dementia	IV	3/3	NL
92-024	89	M	AD dementia	IV	3/3	NL
99-064	89	M	AD dementia	VI	3/3	NL
A197/88	81	M	AD dementia	V-VI	3/3	UK
A005/96	89	M	AD dementia	-	3/3	UK
A012/96	89	M	AD dementia	V-VI	3/3	UK
A013/96	85	M	AD dementia	V-VI	3/3	UK
A342/96	67	M	AD dementia	V-VI	3/3	UK
A277/97	89	M	AD dementia	V-VI	3/3	UK
A065/02	82	M	AD dementia	V-VI	3/3	UK
A093/97	91	M	AD dementia	V-VI	4/4	UK
A200/97	74	M	AD dementia	-	4/4	UK
A213/94	81	F	AD dementia	III-IV	3/3	UK
A335/94	72	F	AD dementia	V-VI	3/3	UK
A125/95	88	F	AD dementia	V-VI	3/3	UK
A044/96	84	F	AD dementia	V-VI	3/3	UK
A097/96	90	F	AD dementia	V-VI	3/3	UK
A291/96	60	F	AD dementia	V-VI	3/3	UK
A407/96	75	F	AD dementia	V-VI	3/3	UK
A025/97	80	F	AD dementia	V-VI	3/3	UK

Journal of Cell Science • Supplementary information

A026/98	80	F	AD dementia	V-VI	3/3	UK
A028/98	70	F	AD dementia	V-VI	3/3	UK
A207/98	91	F	AD dementia	V-VI	3/3	UK
A133/99	92	F	AD dementia	III-IV	3/3	UK
A053/95	76	F	AD dementia	V-VI	4/4	UK
A240/95	75	F	AD dementia	V-VI	4/4	UK
A366/95	79	F	AD dementia	V-VI	4/4	UK
A073/96	89	F	AD dementia	V-VI	4/4	UK
A133/97	92	F	AD dementia	-	4/4	UK
A053/98	89	F	AD dementia	V-VI	4/4	UK

Table S3. Patients examined by immunohistology

**Case No.**	**Age**	**Sex**	**Post-mortem interval (hrs)**	**Clinical diagnosis**	**Thal Phase**	**Braak stage**	**CERAD** neuritic plaque score	**AD** neuropathological change
1	71	M	12	AD dementia	4	V-VI	3	A3, B3, C3
2	75	M	6	AD dementia	4	V-VI	2	A3, B3, C2
3	82	F	24	AD dementia	4	V-VI	3	A3, B3, C3

Neuropathological assessments: All cases were extensively characterized according to the routine protocol for neurodegenerative diseases. Neuropathological diagnosis of AD was made following the NIA-AA criteria including Thal phasing for Aβ load, Braak-and- Braak-staging for NFTs, and CERAD neuritic plaque score to assess the density of neuritic plaques. (Clin Pathol. 2019 Nov;72(11):725-735;. Neuropathology of Neurodegenerative Diseases. (2014). In G. Kovacs (Ed.), Neuropathology of Neurodegenerative Diseases: A Practical Guide (pp. I-Ii). Cambridge: Cambridge University Press).

Table S4. Neurons

The complete data set of proteins identified in the surface proteome of wild-type and sortilin-deficient primary neurons is provided as an Excel file.

Click here to download Table S4

Table S5. Astrocytes

The complete data set of proteins identified in the surface proteome of wild-type and sortilin-deficient primary astrocytes is provided as an Excel file.

Click here to download Table S5
