# Supplementary material for: ApoE4 disrupts interaction of sortilin with fatty acid-binding protein 7 essential to promote lipid signaling
Source: J Cell Sci. 2021 Oct 21;134(20):jcs258894. doi: 10.1242/jcs.258894 (PMC8572006; doi:10.1242/jcs.258894)
Supplement: Reviewer comments [file joces-134-258894_review_history.pdf]

ApoE4 disrupts interaction of sortilin with fatty acid-binding protein 7 essential to promote lipid signaling

Antonino Asaro, Rishabhdev Sinha, Magda Bakun, Oleksandra Kalnytska, Anne-Sophie Carlo-Spiewok, Tymon Rubel, Annemieke Rozeboom, Michal Dadlez, Bozena Kaminska, Eleonora Aronica, Anna Malik and Thomas Willnow
DOI: 10.1242/jcs.258894

Editor: Giampietro Schiavo

Review timeline

Original submission:	10 May 2021
Editorial decision:	1 July 2021
First revision received:	14 September 2021
Accepted:	16 September 2021

Original submission

First decision letter

MS ID#: JOCES/2021/258894

MS TITLE: ApoE4 disrupts interaction of sortilin with fatty acid-binding protein 7 essential to promote lipid signaling

AUTHORS: Antonino Asaro, Rishabhdev Sinha, Magda Bakun, Oleksandra Kalnytska, Anne-Sophie Carlo-Spiewok, Tymon Rubel, Annemieke Rozeboom, Michal Dadlez, Bozena Kaminska, Eleonora Aronica, Anna Malik, and Thomas Willnow
ARTICLE TYPE: Research Article

We have now reached a decision on the above manuscript.

To see the reviewer's report and a copy of this decision letter, please go to: https://submit-jcs.biologists.org and click on the 'Manuscripts with Decisions' queue in the Author Area. (Corresponding author only has access to reviews.)

As you will see, the reviewer and a member of the Editorial Board gave favourable reports but raised some critical points that will require amendments to your manuscript. The finding that sortilin and ApoE regulate the neuronal degradation of FABP7 is particularly important and should be further stressed in the manuscript and abstract. I hope that you will be able to make these and other changes suggested by the reviewer, because I would like to be able to accept your paper.

We are aware that you may be experiencing disruption to the normal running of your lab that makes experimental revisions challenging. If it would be helpful, we encourage you to contact us to discuss your revision in greater detail. Please send us a point-by-point response indicating where you are able to address concerns raised (either experimentally or by changes to the text) and where you will not be able to do so within the normal timeframe of a revision. We will then provide further guidance. Please also note that we are happy to extend revision timeframes as necessary.

© 2021. Published by The Company of Biologists under the terms of the Creative Commons Attribution License (https://creativecommons.org/licenses/by/4.0/).

Please ensure that you clearly highlight all changes made in the revised manuscript. Please avoid using 'Tracked changes' in Word files as these are lost in PDF conversion.
I should be grateful if you would also provide a point-by-point response detailing how you have dealt with the points raised by the reviewers in the 'Response to Reviewers' box. Please attend to all of the reviewers' comments. If you do not agree with any of their criticisms or suggestions please explain clearly why this is so.

Reviewer 1

Advance summary and potential significance to field

Asaro et al. have submitted a well written, novel and interesting manuscript showing that sortilin, the receptor for neuronal uptake of ApoE, binds and stabilizes neuronal FABP7, an intracellular carrier for PUFA in the brain that regulates lipid-dependent gene transcription. They show that this complex is disrupted in ApoE4 neurons, destabilizing FABP7 and impairing lipid signaling similar to what the authors found in ApoE4/4 AD patient brain. I would recommend publication of this manuscript in JCS following revision.

Comments for the author

Please find my observations and suggested revisions below.
Asaro describes the use of an unbiased proteome screens in mouse primary neurons and glia to uncover a relationship between sortilin with fatty acid-binding protein 7 (FABP7). Comparing biotinylated surface proteins in primary neurons from wt or sortilin knockout (KO) mice, the authors found that FABP7 was increased in the surface proteome of KO neurons, suggesting a dysregulation of cellular distribution or degradation of FABP7. This was not observed in primary astrocytes.

The authors then went on to overexpress myc-tagged FABP7 with sortilin in CHO cells, confirming a physical association between sortilin and FABP7 using co-immunoprecipitation analysis, an interaction that was not influenced by the presence of ApoE3 or ApoE4. FABP7 and sortilin copurified in the TGN, the cell surface fraction, and in early endosomes (which may ultimately reflect endolysosomal clearance or secretion of FABP7 and sortilin). Interestingly, FABP7 levels were significantly higher in cells expressing sortilin; sortilin may therefore specifically stabilize (increase the half-life of) the FABP7 protein while having no impact on GFP control levels; this may suggest that sortilin may reduce endolysosomal clearance or secretion of FABP7.

The authors then moved to whole animal experiments. They found that FABP7 total brain levels were higher in wt. vs sortilin KO ApoE3 mice, while they were unchanged in ApoE4 mice, although levels in both ApoE4 wt and sortilin KO looked similar to the ApoE3 sortilin KO levels – this may reflect that sortilin can stabilize FABP7 in the presence of ApoE3 but not in the presence of ApoE4. This may suggest that sortilin is effective to block clearance of FABP7 in ApoE3 but not ApoE4 mice, where the wt basal levels of FABP7 are lower than in ApoE3.

In human AD brain, patients with ApoE3/3 had higher FABP7 protein levels than 4/4 patients, suggesting that the presence of ApoE4 increases clearance of FABP7.
In primary mouse neurons, FABP7 levels were reduced in sortilin KO ApoE3 (Figure 6C). In ApoE4 primary neurons there is no difference FABP7 levels between wt and sortilin KO. It is difficult to tell whether levels of FABP7 in wt ApoE3 and wt ApoE4 are similar (as the FABP7 exposures suggest),

as they are not on the same western. Sortilin KO had no effect on FABP7 levels in astrocytes from ApoE3 or E4 primary cells, but again it is difficult to compare levels between E3 and E4 FABP7 because they are not on the same gel. It appears that FABP7 levels may be higher in wt ApoE4 astrocytes than wt ApoE3 astrocytes (Figure S1), but to compare they must be on the same western blot. From the author's data, it appears the stabilizing effect of sortilin on ApoE3 neuron levels of FABP7 is reduced by ApoE4 and also reduced in astrocytes. I would like to see levels of sortilin and of FABP7 calculated as a % of ApoE3 wt with all samples run on the same gel/western.

The authors found that treatment of CHO-S/F cells with conditioned medium from HEK293 cells secreting human ApoE3 or ApoE4 showed the enhanced degradation of FABP7, and sortilin-FABP7

© 2021. Published by The Company of Biologists under the terms of the Creative Commons Attribution License (https://creativecommons.org/licenses/by/4.0/).

complex perinuclear localization the typical localization of lysosomes. This may suggest that the reduced levels of FABP7 in ApoE4 cells are the result of its enhanced lysosomal degradation. This is fascinating and perhaps should be investigated, using Bafilomycin to inhibit the lysosome to document FABP7 autophagic degradation, by both western analysis and PLA/immunohistochemistry. The localization of sortilin-FABP7 in early endosomes and recycling endosomes in ApoE4 cells is consistent with activated autophagy. It would seem from the data that FABP7 is cleared by autophagy, but sortilin levels do not drop with ApoE4 – there is a disconnect between sortilin and FABP7 and it would seem that sortilin clearance is unaffected by ApoE4 while FABP7 clearance is enhanced. It makes sense that if ApoE4 is enhancing autophagic clearance of FABP7, then the effects of FABP7 on transcription would be reduced in the presence of ApoE4.

I think this paper is interesting, and a revision should be invited. In my view, the major finding in this manuscript is that sortilin and ApoE regulate neuronal degradation of FABP7. When comparing levels of sortilin and FABP7 ApoE3 vs. ApoE4 experiments, levels should be normalized to % of wt ApoE3 and run on the same gel, because it is difficult to compare results when separate gels are run. It would be interesting to know whether the ApoE and FABP7 are cleared together by the lysosome – in this manuscript it appears levels of FABP7 and ApoE4 are reduced in ApoE4 tissue, while sortilin levels are unchanged (figure 6BC) – does sortilin stabilize the ApoE3/FABP7 complex, and is it already destabilized by ApoE4? The endosomal and peri-nuclear localization suggests activated autophagy of FABP7 in ApoE4 cells. In cell culture, this can be investigated through blockage of the lysosome with ammonium chloride/leupeptin or with bafilomycin. The greatest genetic risk factor for late onset Alzheimer's disease is ApoE4. Recently chaperone-mediated autophagy (CMA) has been shown to be dysregulated in Alzheimer's disease particularly in neurons, but not in glia (PMID: 33891876). Sortilin deficiency impacts stability of FABP7 in neurons but not astrocytes, and the mechanism of FABP7 perinuclear/endosomal localization and clearance are consistent with its lysosomal degradation.

Might differences in autophagy between neurons and astrocytes explain cell type specificity for FABP7 stability?

First revision

Author response to reviewers' comments

RESPONSE TO REVIEWER 1:

My co-authors and I greatly appreciate the positive feedback by this reviewer and his/her helpful comments how to further improve our study. We are particularly excited by the hypothesis that cell type-specific mechanisms of protein quality control (i.e., chaperone-mediated autophagy) may explain the distinct stabilities of FABP7 in neurons versus astrocytes identified in our work. This interesting concept has now been discussed in our manuscript as detailed below.

Major point 1:
"´When comparing levels of sortilin and FABP7 ApoE3 vs. ApoE4 experiments, levels should be normalized to % of wt ApoE3 and run on the same gel, because it is difficult to compare results when separate gels are run."

We now completely revised our quantitative analysis of FABP7 levels by running samples of all four genotypes on the same protein gels. As correctly stated by this reviewer, this setup permits us to not only evaluate the impact of sortilin (as in the original manuscript) but also of apoE genotype on FABP7 levels. Given the 3 months period for revision and the large number of mouse brains (n=58) used for our prior study, we here focus on re-analysis of FABP7 levels in primary neurons and astrocytes as these models enable dissection of cell type-specific mechanisms of apoE and sortilin actions (as compared to full brain analyses).

In the revised manuscript, studies in primary cell cultures fully corroborated the relevance of sortilin and of apoE3 for control of neuronal levels of FABP7 by documenting significantly higher

© 2021. Published by The Company of Biologists under the terms of the Creative Commons Attribution License (https://creativecommons.org/licenses/by/4.0/).

levels of FABP7 in E3/WT as compared to E3/KO neurons (Fig. 6C-D). This beneficial effect of sortilin on FABP7 levels is lost with apoE4 as levels of FABP7 are significantly lower in E4/WT as compared to E3/WT neurons (Fig. 6C-D). By contrast, levels of the FABP7 are not impacted by sortilin or apoE genotype in primary astrocytes (Fig. S1C- D).

Taken together, the findings confirmed that stability of FABP7 is controlled by sortilin, an activity specific to neurons and not seen in astrocytes. This protective activity is obviously lost in E3 mice lacking sortilin (E3/KO). However, it is also lost in wild-type neurons in the presence of apoE4 (E4/WT) as this apoE variant disrupts sortilin sorting and renders neurons essentially deficient for receptor activity (Asaro et al., Alzheimer & Dement 2020). It is unclear why levels of FABP7 in E4 neurons increase in the absence of sortilin (compare E4/WT with E4/KO; Fig. 6D). Possibly, this phenomenon results from a secondary compensatory mechanism as suggested by the tendency for increased *Fabp7* transcript levels in E4/KO neurons as compared to all other genotype groups (Fig. 6G, p=0.1028). We now included these considerations in the discussion section (page 11, line 11 from top).

Major point 2:
"It would be interesting to know whether the ApoE and FABP7 are cleared together by the lysosome – in this manuscript it appears levels of FABP7 and ApoE4 are reduced in ApoE4 tissue, while sortilin levels are unchanged (figure 6BC) – does sortilin stabilize the ApoE3/FABP7 complex, and is it already destabilized by ApoE4? In cell culture, this can be investigated through blockage of the lysosome with ammonium chloride/leupeptin or with bafilomycin."

This reviewer raises an exciting hypothesis that escaped our attention, namely a role for lysosomal (autophagosomal) catabolism in control of FABP7 levels; and the impact that apoE4 may have on this process in neurons. As suggested, we tested this hypothesis by treating CHO cells expressing FABP7 and sortilin with apoE4 in the absence or presence of the lysosomal inhibitor bafilomycin. In line with the above hypothesis, blockade of lysosomal catabolism partially rescued the levels of FABP7 in CHO cells treated with apoE4 (figure below). Unfortunately, this effect did not reach statistical significance in a total of three replicate experiments (p=0.13). Thus, while these data provide first evidence for the involvement of lysosomes in neuronal catabolism of FABP7, we strongly feel that many more studies may be required to solidify this hypothesis; studies that exceed the scope and the time line envisioned for revision of this study. Still, we now included this hypothesis in the discussion section to raise awareness for this interesting concept (page 12, line 15 from below).

In addition, we include new data showing that levels of sortilin in neurons are not impacted by apoE4 (Fig. 6E), supporting this reviewer's assumption of distinct mechanisms controlling stability of sortilin and FABP7 in neurons.

NOTE*: We have removed unpublished data that had been provided for the referees in confidence.*

Figure: CHO cells stably expressing sortilin and FABP7 (CHO-S/F) were treated for 24 h with conditioned medium containing 5 µg/ml apoE4 and then for 6 h in the absence (DMSO as solvent control) or the presence (BAF) of 50 nM bafilomycin. Thereafter, levels of FABP7 were determined in cell lysates using western blot analysis and densitometric scanning of replicate blots (n=9 replicates per condition from 3 independent experiments). The statistical significance of differences between treatment conditions were determined using Student's *t* test (p=0.13).

Second decision letter

MS ID#: JOCES/2021/258894

MS TITLE: ApoE4 disrupts interaction of sortilin with fatty acid-binding protein 7 essential to promote lipid signaling

© 2021. Published by The Company of Biologists under the terms of the Creative Commons Attribution License (https://creativecommons.org/licenses/by/4.0/).

AUTHORS: Antonino Asaro, Rishabhdev Sinha, Magda Bakun, Oleksandra Kalnytska, Anne-Sophie Carlo-Spiewok, Tymon Rubel, Annemieke Rozeboom, Michal Dadlez, Bozena Kaminska, Eleonora Aronica, Anna Malik, and Thomas Willnow
ARTICLE TYPE: Research Article

I am happy to tell you that your manuscript has been accepted for publication in Journal of Cell Science, pending standard ethics checks.

© 2021. Published by The Company of Biologists under the terms of the Creative Commons Attribution License (https://creativecommons.org/licenses/by/4.0/).
